# The impact of cognitive reserve on delayed neurocognitive recovery after major non-cardiac surgery: an exploratory substudy

**DOI:** 10.3389/fnagi.2023.1267998

**Published:** 2023-11-23

**Authors:** Elena Kainz, Neelke Juilfs, Ulrich Harler, Ursula Kahl, Caspar Mewes, Christian Zöllner, Marlene Fischer

**Affiliations:** ^1^Department of Anesthesiology, University Medical Center Hamburg-Eppendorf, Hamburg, Germany; ^2^Department of Intensive Care Medicine, University Medical Center Hamburg-Eppendorf, Hamburg, Germany

**Keywords:** delayed neurocognitive recovery, postoperative cognitive dysfunction, perioperative neurocognitive disorders, cognitive reserve, oncological surgery

## Abstract

**Introduction:**

Delayed neurocognitive recovery is a common and severe complication after surgery and anesthesia with an adverse impact on daily living, morbidity, and mortality. High cognitive reserve may mitigate the development of delayed neurocognitive recovery, however, supporting data is lacking. We aimed to assess the association between cognitive reserve and delayed neurocognitive recovery in the early postoperative period.

**Methods:**

This is a substudy of two prospective observational studies. Adult patients undergoing elective major non-cardiac surgery, who were fluent in German, were eligible for study participation. Patients with any pre-existing central nervous system disorders were excluded. Cognitive reserve was assessed using the Cognitive Reserve Index questionnaire. Delayed neurocognitive recovery was defined as a decline in cognitive function compared with baseline assessments and was evaluated with a battery of neuropsychological tests on the day of hospital admission and between day three post procedure and before hospital discharge.

**Results:**

A total of 67 patients with a median age of 67 [IQR: (63–73)] years were included in our analysis. We found delayed neurocognitive recovery in 22.4% of patients. There was a significant association between Cognitive Reserve Index questionnaire total score and the occurrence of delayed neurocognitive recovery in the early postoperative period [OR = 0.938, (95% CI, 0.891; 0.988), *p* = 0.015].

**Conclusion:**

Higher cognitive reserve in elderly patients undergoing major non-cardiac surgery decreases the risk for subsequent delayed neurocognitive recovery in the early postoperative period.

## 1 Introduction

Perioperative neurocognitive disorders – including delayed neurocognitive recovery (DNCR) - are common and severe complications after surgery and anesthesia (Evered et al., 2018). Occurring until 30 days after surgery and anesthesia, DNCR is defined as a new impairment in cognition (Evered et al., 2018), which has been observed in up to 40% of adult patients at hospital discharge after major non-cardiac surgery (Monk et al., 2008). Cognitive decline may be subtle and include deficits in memory, attention, concentration, information processing, and executive function (Monk et al., 2008; Monk and Price, 2011; Evered et al., 2018).

DNCR can be conceptualized as a lack of cognitive resilience in the face of perioperative stress with an adverse impact on quality of life, ability to work, and an increased risk for long-term cognitive decline and mortality after surgery (Monk et al., 2008; Steinmetz, et al., 2009; Nadelson et al., 2014; Berger et al., 2015; Heyer et al., 2015). Previous research has shown that age, comorbid diseases like diabetes and pre-existing cognitive impairment may predispose patients to DNCR (Kline, et al., 2012; Feinkohl et al., 2017a; Yang et al., 2022). However, considering the high prevalence of DNCR and the negative impact associated with it, detailed knowledge on well-established risk factors and therefore, strategies for minimizing risk are lacking. Individual cognitive trajectories after surgery and anesthesia are highly heterogeneous. Data from observational studies suggest that postoperative cognitive function may be determined by preoperative cognitive capacities and a higher level of education (Nadelson et al., 2014; Feinkohl et al., 2017b).

The theoretical construct of cognitive reserve (CR) has been proposed to account for observed discrepancies between pathology or age-related changes and its expected clinical outcome or deficit (Stern, 2009; Kartschmit et al., 2019). Thus, the concept of CR describes the capacity of the brain to mitigate clinical manifestations of a neurodegenerative process or aging.

Stern et al. recently published a framework for defining CR and related concepts to facilitate comparability and communication across investigators. CR is defined as a property of the brain – i.e. multiple potential mechanisms to help cope with or compensate for brain changes and the consequences of brain injury or disease - that allows for cognitive performance that is better than expected given the degree of life-course related structural alterations, brain injury or disease. The extent of CR can be influenced by multiple factors, operating at various points or continuously across lifespan (Stern et al., 2023).

The extent of CR has been linked to the performance of specific intellectual and cognitive activities throughout an individual’s lifespan (Stern, 2003; Stern, 2009; Kartschmit et al., 2019). Surrogates of CR (i.e. level of education, socioeconomic status and pre-morbid cognitive ability) are associated with cognitive impairment in older age (Zeki Al Hazzouri et al., 2011; Meng and D'Arcy, 2012; Feinkohl et al., 2017b). Importantly, a higher CR was found to have protective effects in conditions such as Alzheimer’s disease, Parkinson’s disease, traumatic brain injury and multiple sclerosis (Rami et al., 2011; Sumowski et al., 2013; Hindle et al., 2014; Martins Da Silva et al., 2015; Ciccarelli et al., 2018; Kartschmit et al., 2019). With a lower CR as a predictor of age- and neurodegenerative-related cognitive impairment, it seems plausible to expect an association between preoperative CR and DNCR. We hypothesized that cognitive trajectories after major non-cardiac surgery in the early postoperative period are affected by individual CR.

## 2 Methods

### 2.1 Ethical approval

Ethical approval was obtained by the local ethics committee at the Hamburg Chamber of Physicians (protocol numbers PV4782 and PV4771, approved on September 2, 2014). Written informed consent was obtained from all patients prior to participation.

### 2.2 Study design, setting, and population

The present analysis represents a substudy that includes subsets of patients from two prospective observational studies. The studies were primarily designed to (1) compare pre- and postoperative cognitive function between robot-assisted radical prostatectomy and open retropubic prostatectomy (Beck et al., 2020); (2) assess the association between intraoperative cerebrovascular autoregulation and DNCR after major non-cardiac surgery (unpublished data). Data were collected between 2015 and 2018 at the Department of Anesthesiology, University Medical Center Hamburg-Eppendorf, Germany. Patients aged over 18 years undergoing elective major non-cardiac surgery with a duration >120 min with invasive arterial pressure monitoring were eligible for study participation. Patients were required to have excellent knowledge (language proficiency level C2) of the German language to perform the verbal components of the neuropsychological assessments. Exclusion criteria were any preexisting central nervous system disorders, including cerebrovascular and neurodegenerative disease. Pre-anesthesia visits and electronic medical records were used to screen for eligible patients.

### 2.3 Cognitive reserve and psychometric assessment

We used the Cognitive Reserve Index questionnaire (CRIq) developed by Nucci et al. to measure the quantity of CR accumulated through a lifespan (Nucci et al., 2012). The CRIq includes demographic data and addresses 20 items that are grouped into education (years of education plus vocational training courses), working activity (adulthood professions divided in five different levels), and leisure time activities (cognitively stimulating occupations carried out during leisure time) according to the number of years and frequency of practice. Each of the three domains is recorded in a subscore (Nucci et al., 2012). The CRIq total score is the average of the three subscores and can be classified into five levels: Low (<70), Medium-low (70–84), Medium (85–114), Medium-high (115–130) and High (>130), with a higher CRIq total score indicating a higher CR (Nucci et al., 2012).

Preoperative Mini-Mental Status Examination was performed to screen for pre-existing dementia or mild cognitive impairment (Folstein et al., 1975). Additionally, we used the Cognitive Failures Questionnaire to evaluate the type and frequency of self-reported cognitive failures in everyday life (Broadbent et al., 1982).

We defined DNCR as a decline of cognitive function compared with baseline assessments. Therefore, cognitive function was evaluated twice. Baseline cognitive function was assessed on the day of hospital admission and postoperative cognitive function was evaluated between day three post-procedure and before hospital discharge. Each assessment included a battery of four neuropsychological tests, as reported in detail previously (Beck et al., 2020; Kahl et al., 2022). Briefly, we used the German version of the California Verbal Learning Test to evaluate verbal learning and memory (Testzentrale, Göttingen, Germany) (Niemann et al., 2008), the Trail Making Test A and B to evaluate executive function (Reitan, 1958; Rodewald et al., 2012), the Grooved Pegboard Test to assess visuomotor skills (Liu et al., 2021) (Lafayette Instrument Company, Lafayette, IN), and the Digit Span Forward task to assess attention for the pre- and postoperative assessment of multiple domains of cognitive function. The following subcategories of the California Verbal Learning Test were used in the final analysis: total free recall, learning slope, pro- and retroactive interference, retention of information over short and longer intervals, total number of intrusions, recognition discriminability, free and cued recall (Niemann et al., 2008). In ten patients we applied the Montreal Cognitive Assessment and omitted the California Verbal Learning Test (Nasreddine et al., 2005).

*Z*-scores were calculated as the difference between the pre- and postoperative psychometric test results for each patient and divided by the baseline standard deviation. Combined z-scores were calculated as the sum of *z*-scores for the various tests divided by the standard deviation for normative data z-scores (Tombaugh, 2004; Monaco et al., 2013). DNCR was defined as either a z-score above 1.96 or below −1.96 in the Montreal Cognitive Assessment or at least two subcategories of the California Verbal Learning Test plus one other test (Trail Making Test, Grooved Pegboard Test or Digit Span Forward task) or a combined z-score above 1.96 (Rasmussen, et al., 2001). The definition of DNCR used in the present study is based on the recommendations of Rasmussen et al. and the approach chosen in the ISPOCD1 study (Moller et al., 1998; Rasmussen, et al., 2001). Rather than using the SD of normative values Rasmussen et al. suggested to use the SD from a control group. Since our study was not designed to compare surgical patients to healthy controls not undergoing surgery, we used normative data from age-matched cohorts instead (Trites, 1977; Tombaugh, 2004; Monaco et al., 2013). One essential advantage of the aforementioned method is that calculation can be based on any number of assessments. Thus, a relevant decline in neuropsychological test performance can either comprise a global deterioration of cognitive performance or a severe deterioration in few single tests (Rasmussen, et al., 2001). In addition to the definition of DNCR mentioned above, we calculated a summarized z-score, using the sum of z-scores without standardization to normative data.

Psychometric assessments were performed by a team of specially trained medical professionals. Each patient was tested by the same psychometrician pre- and postoperatively in a quiet room with only the patient and the examiner present. A list of assessments performed throughout the perioperative period is presented in Supplementary material 1.

### 2.4 Surgery and anesthesia

General anesthesia was administered according to our institutional standard operating procedures. Epidural anesthesia was performed in patients undergoing solid tumor resection (other than radical prostatectomy), if there was no contraindication for neuraxial anesthesia. Sufentanil (0.3–0.5 μg/kg) and propofol (2–3 mg/kg) were used for anesthesia induction, followed by neuromuscular blockade with rocuronium (0.6 mg/kg) to facilitate endotracheal intubation. Sevoflurane-sufentanil (age-adjusted MAC 0.8–1.2) or propofol-sufentanil (4–8 mg/kg/h) was used for anesthesia maintenance, targeting a bispectral index of 30–40 (bispectral index monitor, BIS™, Medtronic GmbH, Meerbusch, Germany). Arterial blood pressure was measured continuously with an arterial catheter placed in the radial or femoral arteries. Normothermia was maintained using a forced-air warming system throughout the entire procedure.

### 2.5 Statistical analysis

Continuous data are presented as medians with interquartile ranges, and categorial data are presented as frequencies with percentages. For group comparisons (no DNCR vs. DNCR) the Mann–Whitney U-test (continuous variables), Chi-square test, or Fisher’s exact test (categorical variables) were used as appropriate. The association between CR and DNCR in the early postoperative period was analyzed with binary logistic regression. The independent variable of primary interest (CRIq total score) and clinically relevant variables (age, American Society of Anesthesiologists [ASA] physical status, epidural anesthesia, total dose of sufentanil, duration of surgery) were included in the multivariable model with DNCR as the dependent variable. Independent variables were eliminated stepwise backwards to obtain the final model. Continuous variables were visually assessed for normal distribution using histograms. The variables ‘total dose of sufentanil’ and ‘duration of surgery’ were logarithmically transformed for the logistic regression analysis. We performed a sensitivity analysis excluding patients, who underwent cognitive assessments with the Montreal Cognitive Assessment instead of the California Verbal Learning Test. For the sensitivity analysis, logistic regression was modeled in analogy with the main analysis. To address within-subject changes without standardization to normative data, we performed another sensitivity analysis with the summarized z-score as dependent variable. Linear regression was used to assess the association between independent variables and the summarized z-score. Independent variables were chosen by clinical relevance in analogy to the main model that included the binary variable ‘DNCR’ as dependent variable. ‘CRIq total score’ as the independent variable of primary interest was forced into the model, while the remaining independent variables were eliminated stepwise backwards. We used SPSS Statistics 27 (IBM Corporation, Armonk, New York) for statistical analyses.

## 3 Results

### 3.1 Study population and patient characteristics

**Figure 1:**
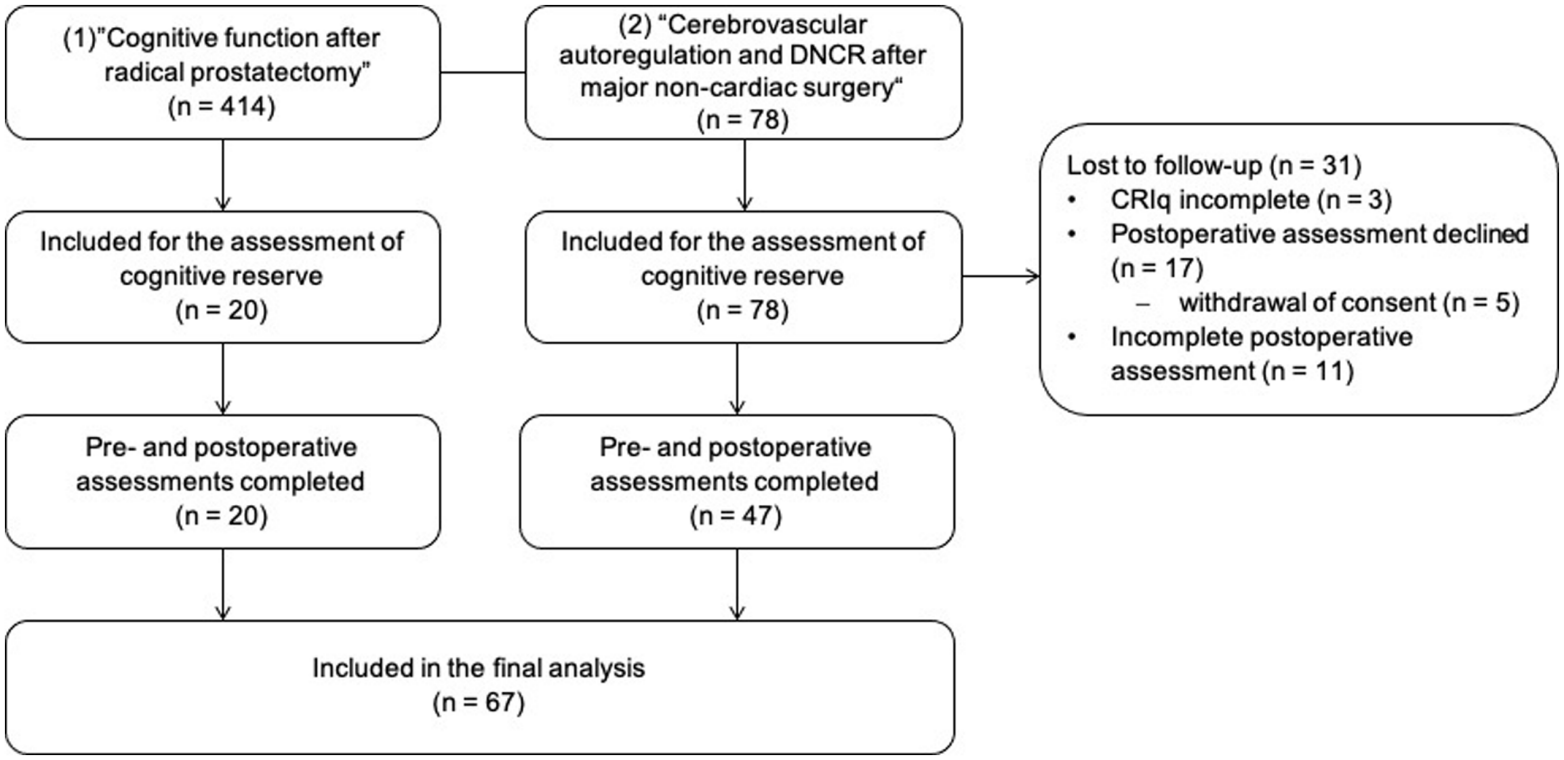
Flow of participants throughout the study. The study population consisted of subsets of patients from two prospective observational studies that were designed to (1) compare pre- and postoperative cognitive function between patients with robot-assisted and with open retropubic radical prostatectomy, and to (2) assess the association between intraoperative cerebrovascular autoregulation and delayed neurocognitive recovery (DNCR). CRIq, Cognitive Reserve Index questionnaire.

Complete assessments for CR and DNCR were available from 67 patients, who were included in the final analysis (Figure 1). Patient characteristics stratified by DNCR status are shown in Table 1. Our study population consisted primarily of elderly patients with a median age of 67 years with no significant difference between groups [no DNCR: 67 (63–73) vs. DNCR: 67 (63–74)]. The majority of patients were male (71.6%, *n* = 48/67) and fulfilled the criteria for level II or III in the ASA physical status classification system (95.5%; *n* = 64/67). There was no relevant imbalance in the preoperative Cognitive Failures Questionnaire or Mini-Mental Status Examination between patients with and without DNCR. Variables related to surgery and anesthesia are listed in Table 1. Thirty-one patients were lost to follow-up and did not complete the postoperative neuropsychological assessments for various reasons (Figure 1). Demographic and clinical characteristics of these patients and a comparison of characteristics (lost to follow-up vs. complete datasets) are listed in Supplementary material 2.

**Table 1:** Baseline characteristics, education, baseline psychometric assessment and perioperative characteristics by the presence of delayed neurocognitive recovery

	No DNCR (*n* = 52)	DNCR (*n* = 15)	*p*
*Age (years)*	67 (63-73)	67 (63-74)	0.898
*BMI (kg/m^2^)*	25.5 (23.4-28.0)	25.1 (23.1-28.3)	0.668
*Sex*			0.352
Female	14 (26.9)	6 (40.0)	
Male	38 (73.1)	9 (60.0)	
*Obesity (BMI ≥30)*	7 (13.5)	2 (13.3)	1.000
*Arterial hypertension*	25 (48.1)	9 (60.0)	0.416
*Dyslipoproteinemia*	12 (23.1)	4 (26.7)	1.000
*Diabetes mellitus*	6 (11.5)	1 (6.7)	0.686
*Positive smoking status*	9 (17.3)	5 (33.3)	0.277
*OSAS*	3 (5.8)	0 (0.0)	0.585
*ASA physical status classification*			0.542
I	4 (7.7)	0 (0.0)	
II	24 (46.2)	6 (40.0)	
III	23 (44.2)	9 (60.0)	
IV	1 (1.9)	0 (0.0)	
*Education*			0.552
< high school	33 (63.5)	11 (73.3)	
≥ high school	19 (36.5)	4 (26.7)	
**Baseline psychometric assessment**			
CFQ	22 (15-29)	21 (17-30)	0.804
MMSE	28 (26-29)	28 (27-28)	0.745
**Perioperative characteristics**			
*Duration of surgery (min)*	212 (153-305)	190 (141-295)	0.899
*Estimated blood loss (ml)*	325 (100-600)	150 (0-700)	0.225
*Epidural anesthesia*	33 (60.0)	12 (80.0)	0.140
*Sufentanil (total amount, μg)*	80 (60-100)	70 (50-95)	0.270
*Type of anesthesia*			0.580
Inhalational anesthesia	49 (94.2)	13 (86.7)	
Total intravenous anesthesia	3 (5.8)	2 (13.3)	
*Surgical discipline*			0.258
General surgery	19 (36.5)	9 (60.0)	
Urology	28 (53.8)	4 (26.7)	
Gynecology	4 (7.7)	2 (13.3)	
Traumatology	1 (1.9)	0 (0.0)	
*Length of hospital stay (days)*	11 (5-17)	10 (6-13)	0.970
*Postoperative assessment (days)*	7 (4-11)	7 (5-12)	0.574

ASA, American Society of Anesthesiologists; BMI, body mass index; CFQ, Cognitive Failures Questionnaire; DNCR, delayed neurocognitive recovery; OSAS, obstructive sleep apnea syndrome. Continuous variables are presented as median with interquartile range. Categorical variables are presented as absolute and relative numbers.

### 3.2 Cognitive reserve and delayed neurocognitive recovery

Signs of DNCR in the early postoperative period were present in 22.4% of patients (*n* = 15/67). Cognitive assessments were performed at a median of 7 days after surgery. Patients diagnosed with DNCR had lower CRIq total scores (113 [109–120] vs. 125 [111–134]) and lower CRIq working subscores [107 (98–116) vs. 116 (104–131)] than patients without DNCR. CRIq education and CRIq leisure time subscores did not differ significantly between groups. Detailed information regarding CR stratified by DNCR status are shown in Table 2.

**Table 2:** Cognitive reserve by the presence of delayed neurocognitive recovery.

	No DNCR (*n* = 52)	DNCR (*n* = 15)	*p*
**CRIq total score**	125 (111-134)	113 (109-120)	0.019
**CRIq education**	113 (104-126)	109 (103-115)	0.268
**CRIq working**	116 (104-131)	107 (98-116)	0.017
**CRIq leisure**	122 (111-131)	112 (105-123)	0.073
**CRIq categories**			0.026
Medium	17 (32.7)	9 (60.0)	
Medium-high	12 (23.1)	5 (33.3)	
High	23 (44.2)	1 (6.7)	

CRIq, Cognitive Reserve Index questionnaire; DNCR, delayed neurocognitive recovery. Continuous variables are presented as median with interquartile range. Categorical variables are presented as absolute and relative numbers.

We found a significant association between CRIq total scores and the occurrence of DNCR in the early postoperative period [OR = 0.938, (95% CI: 0.891; 0.988), *p* = 0.015]. There was no statistical association between age, ASA physical status, epidural anesthesia, total dose of sufentanil, the duration of surgery and DNCR. The first and the final steps of the logistic regression model are presented in Table 3.

**Table 3:** Multivariable logistic regression for the association between clinically relevant variables and delayed neurocognitive recovery (dependent variable).

	OR	95% CI (lower)	95% CI (upper)	*p*
**First step**				
Age (per year increase)	0.980	0.885	1.085	0.697
Sex	1.069	0.245	4.674	0.929
ASA physical status I&II^a^	1.083	0.268	4.373	0.911
Epidural anesthesia^b^	1.792	0.328	9.785	0.501
Sufentanil (per μg increase)	0.094	0.002	5.610	0.257
Duration of surgery	0.454	0.019	10.961	0.627
CRIq total score	0.933	0.880	0.988	0.018
**Last step**				
CRIq total score	0.938	0.891	0.988	0.015

Independent variables were eliminated stepwise backwards.

ASA, American Society of Anesthesiologists; CRIq, Cognitive Reserve Index questionnaire. The variables ‘sufentanil’ and ‘duration of surgery’ were logarithmically transformed to achieve normal distribution.

^a^Reference: ASA III.

^b^Reference: no epidural anesthesia.

### 3.3 Sensitivity analyses

Patients, who underwent assessment with the Montreal Cognitive Assessment were excluded from logistic regression analysis for the association between clinically relevant variables and DNCR. When analyzing only patients, who were tested with the California Verbal Learning Test but not the Montreal Cognitive Assessment, the association between the CRIq total score and DNCR remained significant [OR = 0.935, (95% CI: 0.884; 0.988), *p* = 0.017]. The first and the final steps are presented in Supplementary material 3.

We performed backward stepwise linear regression analysis with the summarized z-score as dependent variable to assess within-subject changes within one statistical model. When using the summarized z-score as dependent variable, we did not find a significant association between the CRIq total score [B = −0.001, (95% CI: −0.014; 0.012), *p* = 0.855] and cognitive function (Supplementary material 4).

## 4 Discussion

In the present study, elderly patients with a lower CRIq total score had a significantly higher risk of DNCR after major non-cardiac surgery. This indicates that a lower CR predisposes for subsequent DNCR in the early postoperative period.

Our finding strengthens the concept of CR as a protective determinant for postoperative cognitive function and our results are in line with previous data evaluating the impact of CR on DNCR. A systematic review and meta-analysis found that patients with a relatively higher level of CR – assessed by education – are at reduced risk for DNCR (Feinkohl et al., 2017b). The level of education is a commonly used proxy indicator for CR. However, it is important to note that educational background alone does not adequately reflect the multifactorial concept of CR (Kartschmit et al., 2019).

Measuring and quantifying CR, i.e., assessing the ability to optimize cognitive performance through recruitment of neuronal networks and/or compensation by alternative cognitive strategies, is challenging (Nucci et al., 2012; Kartschmit et al., 2019). A common approach to indirectly assess CR and its multiple components, is using standardized questionnaires that include the main sociobehavioral proxy indicators of CR such as education, professional occupation, physical and leisure activity, and premorbid intelligence (Nucci et al., 2012; Kartschmit et al., 2019).

To date, there are only few studies evaluating the impact of CR on DNCR using standardized psychometric instruments that combine several main proxy indicators to measure CR as a multifactorial concept. A prospective observational study investigated DNCR after total joint replacement and its relationship with CR (Scott et al., 2017). Similar to our approach, the authors used the CRIq for the evaluation of CR and a battery of cognitive tests to assess DNCR. They found a positive correlation between CR and postoperative cognitive function within a subset of patients who experienced the greatest change (either impairment or improvement) in their cognitive performance (Scott et al., 2017). The results provide some preliminary support for the protective effect of CR on postoperative cognitive recovery, which is in line with our results. This further underlines the importance of standardization, when it comes to the multidimensional assessment of CR, and therefore, allowing comparison of results from different studies.

We found a significant association between CRIq total scores and the occurrence of DNCR. Study participants diagnosed with DNCR scored lower in CRIq total scores and had lower CRIq working subscores compared with patients without DNCR. Interestingly, CRIq education and CRIq leisure time subscores did not differ significantly between groups. This indicates that working experience may be a central component in an individual’s CR accumulated through a lifespan and underlines the importance of assessing the concept of CR as a multicomponent construct rather than solely taking into account the level of education.

A preoperative assessment of CR alongside with baseline evaluation of cognitive function in elderly patients might be useful to identify individuals with a higher susceptibility for postoperative cognitive decline. Screening would be key to better recognition and prevention of perioperative cognitive complications in a growing elderly population undergoing surgery with higher risk for postoperative cognitive decline *per se* (Feinkohl et al., 2017b; Evered et al., 2018; Evered and Silbert, 2018). Routine preoperative neuropsychologic assessment of elderly patients facilitates appropriate preoperative counseling regarding the risk of cognitive decline post intervention. Transparent discussions with patients and caregivers are needed to decide whether the benefits of surgery outweigh the risk of cognitive decline post intervention and its associated socioeconomic and individual impact (Feinkohl et al., 2017b; Brodier and Cibelli, 2021).

For patients at high risk for DNCR, preventive measures are urgently needed. Interestingly, there is data from clinical and experimental studies showing that preoperative cognitive intervention and enrichment of activity reduces the rate of DNCR (Kawano et al., 2015; Saleh et al., 2015). Currently, Butz et al. are conducting a two-arm randomized controlled intervention study that aims to strengthen CR to protect against postoperative neurocognitive disorders after elective cardiosurgical interventions using preoperative, home-based, cognitive training (Butz et al., 2022). Considering that CR might be modifiable (Kartschmit et al., 2019; Stern et al., 2023) and that there might be a preventive effect on the incidence of postoperative cognitive decline through the performance of mentally and physically stimulating activities, the approach of preoperative cognitive training of high-risk patient cohorts seems promising and warrants further investigation.

In our study population of elderly patients who underwent major non-cardiac surgery, the incidence of DNCR in the early postoperative period was 22.4%. Our data is in line with previous on DNCR one week after surgery (Moller et al., 1998). Our findings underscore that DNCR is a perioperative complication of concern from a public health perspective. The lack of clear diagnostic criteria, inconsistencies in diagnosis of DNCR and the heterogeneity of neuropsychological instruments used in previous studies have led to a substantial variability in the prevalence and data regarding perioperative neurocognitive disorders. Interestingly, there was no significant association of CRIq total scores with summarized *z*-scores. These findings emphasize how important it is to include a general deterioration (combined *z*-score) and very severe deficits in few tests (*z*-scores for single test parameters) in DNCR diagnosis. The 2018 definition of “postoperative cognitive dysfunction” as DNCR if present within 30 days after surgery by The Nomenclature Consensus Working Group (Evered et al., 2018) is an important step toward a consistent nomenclature and diagnostic framework for DNCR - even though it is not yet defined by the DSM-5.

### 4.1 Strengths and limitations

Several caveats should be considered when interpreting the findings of our study. First, this is a substudy from two prospective observational single-center studies. Hence, our results are of exploratory nature and should be interpreted with caution due to limited external validity.

Missing data due to incomplete neuropsychological assessments postoperatively may have led to underdiagnosis of DNCR in our study population. Patients, who were lost to follow-up, had a higher BMI, suffered from diabetes more frequently, and had longer surgeries with more extensive blood loss than patients who completed postoperative assessments. Therefore, we may have missed patients with reduced cognitive performance, who refused to undergo postoperative testing to conceal cognitive impairment.

We used the CRIq developed by Nucci et al., which has been shown to provide a reliable assessment of CR. Yet, psychometric properties remain difficult to evaluate considering the lack of a gold standard measurement of CR (Nucci et al., 2012; Kartschmit et al., 2019). Thus, there is a need of high-quality methodological studies assessing the properties of established CR questionnaires including the CRIq, especially regarding content validity, structural validity, and responsiveness (Kartschmit et al., 2019).

Cognitive assessments were performed at a median of seven days postoperatively. There are recommendations that testing for DNCR should not be conducted earlier than seven days after surgery considering the acute effects of surgery and hospitalization confounding cognitive function (Brodier and Cibelli, 2021). However, we chose to perform postoperative assessment before hospital discharge, which was earlier than seven days in some patients.

We did not assess subjective impairment during activities of daily living, which has been recommended by The Nomenclature Consensus Working Group (Evered et al., 2018). This is attributable to the fact that our study was designed before publication of the current recommendations. Importantly, we did not screen for signs of postoperative delirium, the presence of which may have contributed to loss to follow-up and may have compromised the postoperative assessment of cognitive performance.

More than two thirds of our patients were male and without preexisting cognitive impairment in baseline Mini-Mental Status Examination. Thus, our study population consisted mainly of high functioning individuals with a high level of CR. As a consequence, the generalizability of our findings might be limited and should be confirmed in more diverse populations. Of note, the small sample size limits statistical power and may have caused a type II error. However, the strong statistical association between the CRIq and DNCR that is reproduced in the sensitivity analysis points toward an actual effect of the CRIq on DNCR.

To date, only few studies have provided prospective data evaluating CR in the perioperative setting and have used a rigorous methodology for assessing both CR and DNCR. In our study, applying the CRIq as a standardized questionnaire allowed to measure and quantify CR as a complex multifactorial concept. Serial psychometric assessments with a battery of tests were conducted to identify subtle features of cognitive decline in multiple cognitive domains and therefore increasing the accuracy of DNCR diagnosis. Using the aforementioned methods for assessing CR and DNCR is an important strength of our study. Our findings add to the body of evidence on the protective effect of CR and underline the importance of standardization of CR measurements to allow for a better comparability in future studies.

## 5 Conclusion

In a cohort of elderly patients who underwent major non-cardiac surgery, we found a significant association between CR and DNCR in the early postoperative period. Our data suggest that higher CR decreases the risk for subsequent DNCR. The concept of CR should be considered when it comes to minimizing the risk for postoperative cognitive decline. This is especially important in a growing population of elderly patients with a higher susceptibility for perioperative neurocognitive disorders *per se* (Evered and Silbert, 2018). The association between CR and DNCR in the early postoperative period warrants further investigation in future prospective studies including larger samples and a more diverse study population.

## Data availability statement

The datasets presented in this article are not readily available because of the German general data protection regulation. Requests to access the datasets should be directed to MF; mar.fischer@uke.de.

## Ethics statement

Ethical approval was obtained by the local ethics committee at the Hamburg Chamber of Physicians (protocol numbers PV4782 and PV4771, approved on September 2, 2014). Written informed consent was obtained from all patients prior to participation.

## Author contributions

EK: Data curation, Investigation, Writing – original draft, Writing – review & editing. NJ: Investigation, Writing – review & editing. UH: Data curation, Formal analysis, Investigation, Writing – review & editing. UK: Writing – review & editing. CM: Writing – review & editing. CZ: Resources, Supervision, Writing – review & editing. MF: Conceptualization, Data curation, Formal analysis, Investigation, Project administration, Resources, Writing – original draft, Writing – review & editing.
