# Supplementary material for: The impact of cognitive reserve on delayed neurocognitive recovery after major non-cardiac surgery: an exploratory substudy
Source: Front Aging Neurosci. 2023 Nov 23;15:1267998. doi: 10.3389/fnagi.2023.1267998 (PMC10701404; doi:10.3389/fnagi.2023.1267998)
Supplement: Supplementary file 1 [file Data_Sheet_1.PDF]

Supplementary Material

The impact of cognitive reserve on delayed neurocognitive recovery after major non-cardiac surgery: an exploratory substudy

Elena Kainz, Neelke Juilfs, Ulrich Harler, Ursula Kahl, Caspar Mewes, Christian Zöllner, Marlene Fischer*

*** Correspondence:** Marlene Fischer: mar.fischer@uke.de

Supplementary material 1. Psychometric evaluation before and after surgery.

**Preoperative assessments**	**Postoperative assessments**
• Cognitive Reserve: CRIq<br>• Pre-existing cognitive impairment: MMSE<br>• Self-reported cognitive failures: CFQ<br><br>• CVLT/MOCA<br>• Trail Making Test<br>• Grooved Pegboard Test<br>• Digit Span Forward	• CVLT/MOCA<br>• Trail Making Test<br>• Grooved Pegboard Test<br>• Digit Span Forward

Supplementary material 1. Psychometric evaluation before and after surgery. *CFQ* Cognitive Failures Questionnaire, *CRIq* Cognitive Reserve Index questionnaire, *CVLT* California Verbal Learning Test, *MMSE* Mini-mental Status Examination, *MoCA* Montreal Cognitive Assessment.
