# Supplementary material for: The impact of cognitive reserve on delayed neurocognitive recovery after major non-cardiac surgery: an exploratory substudy
Source: Front Aging Neurosci. 2023 Nov 23;15:1267998. doi: 10.3389/fnagi.2023.1267998 (PMC10701404; doi:10.3389/fnagi.2023.1267998)
Supplement: Supplementary file 2 [file Data_Sheet_2.pdf]

Supplementary Material

The impact of cognitive reserve on delayed neurocognitive recovery after major non-cardiac surgery: an exploratory substudy

Elena Kainz, Neelke Juilfs, Ulrich Harler, Ursula Kahl, Caspar Mewes, Christian Zöllner, Marlene Fischer*

*** Correspondence:** Marlene Fischer: mar.fischer@uke.de

Supplementary material 2. Lost to follow-up.

	Complete datasets n = 67	Lost to follow-up n = 26	*p*-value
**Age (years)**	67 (63-74)	69 (64-75)	0.810
**Sex**			0.068
Female	20 (29.9)	13 (50.0)	
Male	47 (70.1)	13 (50.0)	
**BMI (kg/m^2)**	25.4 (23.3-28.1)	27.8 (25.6-29.7)	0.036
**Obesity**	9 (13.4)	5 (19.2)	0.525
**Arterial hypertension**	34 (50.7)	18 (69.2)	0.107
**Dyslipoproteinemia**	16 (23.9)	8 (30.8)	0.496
**Diabetes mellitus**	7 (10.4)	8 (30.8)	0.017
**Current smoking status**	14 (20.9)	10 (38.5)	0.082
**OSAS**	3 (4.5)	0 (0.0)	0.557
**ASA**			0.526
I	4 (6.0)	1 (3.8)	
II	30 (44.8)	8 (30.8)	
III	32 (47.8)	16 (61.5)	
IV	1 (1.5)	1 (3.8)	
**Education**			0.744
<highschool	44 (65.7)	18 (69.2)	
≥highschool	23 (34.3)	8 (30.8)	
**CFQ**	22 (16-30)	16 (9-25)	0.028
**MMSE**	28 (28-29)	28 (27-29)	0.452
**Duration of surgery, min**	203 (150-305)	298 (190-425)	0.006
**Estimated blood loss, ml**	300 (50-700)	675 (100-1500)	0.014
**Epidural anesthesia**	42 (62.7)	19 (73.1)	0.344
**Sufentanil, µg**	75 (55-100)	93 (60-135)	0.072

**Type of anesthesia**			0.136
Inhalational	62 (92.5)	21 (80.8)	
TIVA	5 (7.5)	5 (19.2)	
**Surgical discipline**			0.027
General surgery	28 (41.8)	19 (73.1)	
Urology	32 (47.8)	4 (15.4)	
Gynecology	6 (9.0)	3 (11.5)	
Traumatology	1 (1.5)	0 (0.0)	
**Length of hospital stay, days**	10 (5-16)	13 (6-34)	0.141
**CRIq total score**	119 (110-132)	118 (105-125)	0.288
**CRIq subscore (leisure)**	121 (109-130)	122 (108-128)	0.807
**CRIq subscore (working)**	114 (103-129)	103 (98-122)	0.194
**CRIq subscore (education)**	111 (103-125)	108 (105-111)	0.253

Supplementary material 2. *ASA* American Society of Anesthesiologists, *BMI* Body mass index, *CFQ* Cognitive Failures Questionnaire, *CRIq* Cognitive Reserve Index questionnaire, *DNCR* Delayed neurocognitive recovery, *OSAS* Obstructive sleep apnea syndrome, *TIVA* Total intravenous anesthesia. For group comparisons (lost to follow-up vs. complete datasets) the Mann-Whitney U-test (continuous variables), Chi-square test, or Fisher's exact test (categorical variables) were used as appropriate. Continuous variables are presented as median with interquartile range. Categorical variables are presented as absolute and relative numbers.
