# Supplementary material for: The impact of cognitive reserve on delayed neurocognitive recovery after major non-cardiac surgery: an exploratory substudy
Source: Front Aging Neurosci. 2023 Nov 23;15:1267998. doi: 10.3389/fnagi.2023.1267998 (PMC10701404; doi:10.3389/fnagi.2023.1267998)
Supplement: Supplementary file 3 [file Data_Sheet_3.pdf]

frontiers

Supplementary Material

The impact of cognitive reserve on delayed neurocognitive recovery after major non-cardiac surgery: an exploratory substudy

Elena Kainz, Neelke Juilfs, Ulrich Harler, Ursula Kahl, Caspar Mewes, Christian Zöllner, Marlene Fischer*

*** Correspondence:** Marlene Fischer: mar.fischer@uke.de

Supplementary file 3. Sensitivity analysis.

	OR	95% CI (lower)	95% CI (upper)	*p*
**First step**				
Age (per year increase)	0.941	0.840	1.054	0.293
ASA physical status I&II[a]	0.930	0.208	4.166	0.925
Epidural anesthesia[b]	2.960	0.508	17.255	0.228
Sufentanil (per µg increase)	0.040	0.001	2.548	0.129
Duration of surgery	0.175	0.006	4.893	0.305
CRIq total score	0.937	0.885	0.993	0.027
**Last step**				
Sufentanil (per µg increase)	0.030	0.001	1.333	0.070
CRIq total score	0.935	0.884	0.988	0.017

Supplementary file 3. Multivariable logistic regression for the association between clinically relevant variables and delayed neurocognitive recovery (dependent variable). Variables were eliminated stepwise backwards. Patients, who were tested with the Montreal Cognitive Assessment were excluded from the analysis. *ASA* American Society of Anesthesiologists. The variables 'sufentanil' and 'duration of surgery' were logarithmized to achieve normal distribution. [a]Reference: ASA III. [b]Reference: no epidural anesthesia.
