# Supplementary material for: The impact of cognitive reserve on delayed neurocognitive recovery after major non-cardiac surgery: an exploratory substudy
Source: Front Aging Neurosci. 2023 Nov 23;15:1267998. doi: 10.3389/fnagi.2023.1267998 (PMC10701404; doi:10.3389/fnagi.2023.1267998)
Supplement: Supplementary file 4 [file Data_Sheet_4.PDF]

Supplementary Material

The impact of cognitive reserve on delayed neurocognitive recovery after major non-cardiac surgery: an exploratory substudy

Elena Kainz, Neelke Juilfs, Ulrich Harler, Ursula Kahl, Caspar Mewes, Christian Zöllner, Marlene Fischer*

*** Correspondence:** Marlene Fischer: mar.fischer@uke.de

Supplementary file 4. Within subject changes.

	B	95% CI (lower)	95% CI (upper)	*p*
**First step**				
Age (per year increase)	-0.017	-0.050	0.016	0.307
Sex	-0.010	-0.482	0.462	0.967
ASA physical status I&II[a]	-0.152	-0.561	0.257	0.460
Epidural anesthesia[b]	0.046	-0.420	0.511	0.845
Sufentanil (per µg increase)	-0.364	-1.582	0.853	0.551
Duration of surgery	1.229	0.221	2.236	0.018
CRIq total score	-0.003	-0.017	0.11	0.691
**Last step**				
Duration of surgery	1.146	0.278	2.015	0.011
CRIq total score	-0.001	-0.014	0.012	0.855

Supplementary file 4. Linear regression analysis for the association between clinically relevant variables and summarized z-scores (dependent variable). 'CRIq total score' as the independent variable of primary interest was forced into the model. *ASA* American Society of Anesthesiologists. *CRIq* Cognitive Reserve Index questionnaire. The variables 'sufentanil' and 'duration of surgery' were logarithmized to achieve normal distribution. [a]Reference: ASA III. [b]Reference: no epidural anesthesia.
